# The Sooner, the Better: Neuroprotective Strategies in Fetuses With Congenital Heart Disease

**DOI:** 10.1002/pd.70069

**Published:** 2026-01-26

**Authors:** Maaike Nijman, Mirthe E. M. van der Meijden, Johannes M. P. J. Breur, Raymond Stegeman, Nicolaas J. G. Jansen, Mireille N. Bekker, Manon J. N. L. Benders, Nathalie H. P. Claessens

**Affiliations:** ^1^ Center for Congenital Heart Disease Utrecht Wilhelmina Children's Hospital University Medical Center Utrecht Utrecht the Netherlands; ^2^ Department of Neonatology Wilhelmina Children's Hospital University Medical Center Utrecht Utrecht the Netherlands; ^3^ Department of Pediatrics Beatrix Children's Hospital University Medical Center Groningen Groningen the Netherlands; ^4^ Department of Obstetrics and Gynecology University Medical Center Utrecht Utrecht the Netherlands; ^5^ Department of Pediatrics Wilhelmina Children's Hospital University Medical Center Utrecht Utrecht the Netherlands

**Keywords:** brain development, brain injury, congenital heart disease, fetus, neurodevelopmental outcome, neuroprotection

## Abstract

Congenital heart disease (CHD) is the most frequent congenital malformation at birth and is associated with neurodevelopmental impairments. Alterations in cardiovascular physiology can lead to reduced cerebral blood perfusion and oxygenation, which negatively affects brain growth and maturation. Advanced imaging studies indicate that these aberrations in brain development can manifest as early as in utero, resulting from the inability of the fetal circulatory system to meet the increased metabolic demands of the brain. Fetal brain dysmaturation increases the susceptibility to postnatal brain injury and is related to adverse long‐term neurodevelopmental outcomes throughout childhood. This emphasizes the potential for effective prenatal neuroprotective strategies in fetuses with CHD, as optimization of their intrauterine environment may prevent irreversible neurological damage and minimize long‐term neurodevelopmental comorbidities. This review provides a comprehensive overview of prenatal neuroprotective strategies in fetuses with critical CHD, including in utero therapeutic interventions, prenatal surgical cardiac interventions, and modifiable prenatal and perinatal risk factors.

## Summary

- What is already known about this topic?
  ◦ Fetuses with congenital heart disease are at risk of impaired fetal brain development, which is related to adverse long‐term neurodevelopmental outcomes.
- What does this study add?
  ◦ To optimize neurologic outcomes, we encourage the consideration and targeted management of modifiable risk factors in perinatal care, such as gestational age at birth, maternal and placental health, and prenatal maternal stress exposure.
  ◦ The in‐utero therapeutic interventions and prenatal surgical cardiac interventions studied to date require more research before they can be implemented in standard care regimens.

**Abbreviations**
CHD: congenital heart disease
HLHS: hypoplastic left heart syndrome
LSOL: left‐sided obstructive lesions
MCA‐PI: middle cerebral artery pulsatility index
MH: maternal hyperoxygenation
SVP: single‐ventricle physiology
TGA: transposition of the great arteries

## 1 Introduction

Congenital heart disease (CHD) is the most prevalent birth defect characterized by structural abnormalities of the heart or intrathoracic great vessels [1, 2]. Advances in surgical techniques and perioperative care have led to a substantial decrease in mortality among infants with critical CHD, providing 90% of these children with the prospect of survival into adulthood [3, 4, 5]. However, survivors of critical CHD show adverse long‐term neurodevelopmental outcomes, believed to originate in part from altered fetal cerebral development due to suboptimal fetal circulation [6, 7, 8, 9]. The influence of the in utero environment is increasingly recognized. Fetal imaging studies have shown widespread changes in brain development, with some of these being linked to prenatal factors, such as maternal comorbidities, fetal growth factors, maternal stress exposure, and placental health [10, 11, 12, 13, 14].

**FIGURE 1:**
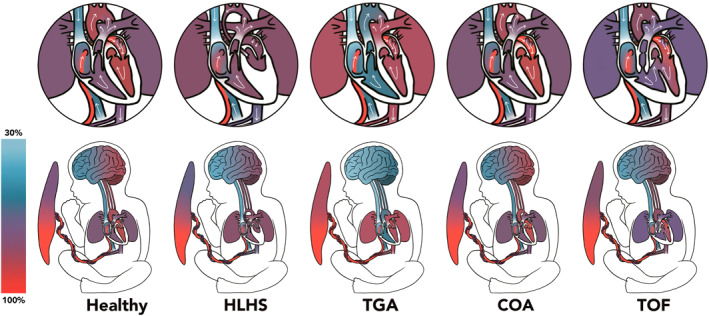
Fetal circulation and oxygenation per CHD subtype. HLHS: hypoplastic left heart syndrome; TGA: transposition of the great arteries; COA: coarctation of the aorta; TOF: tetralogy of Fallot.

The hemodynamic consequences of each CHD subtype have unique effects on fetal cerebral blood flow. In healthy fetuses, oxygenated placental blood flows to the fetal heart via the ductus venosus, where the right‐to‐left shunts (i.e., foramen ovale and ductus arteriosus) ensure oxygenated blood to flow out of the ascending aorta to the systemic circulation, including the brain [7, 15]. Complex cardiac defects include single‐ventricle physiology (SVP) (e.g., hypoplastic left heart syndrome (HLHS)), transposition of the great arteries (TGA), aortic arch anomalies (e.g., coarctation of the aortae), and tetralogy of Fallot [16]. SVP is characterized by underdevelopment of one of the ventricles of the heart, causing diminished ventricular output and dissemination of oxygen‐poor blood throughout the body [7]. In TGA, well‐oxygenated blood from the left ventricle flows to the pulmonary circulation, whereas the cerebral circulation receives largely deoxygenated blood from the right ventricle. As for aortic arch anomalies, left‐ventricular output is compromised due to narrowing or hypoplasia of the aorta, but the blood oxygenation level is largely similar to that of healthy fetuses [17]. Lastly, in tetralogy of Fallot, the main pulmonary artery is narrowed, thereby decreasing right‐ventricular output, and deoxygenated blood shunts from the right into the left ventricle via a septal defect, flowing into the systemic circulation [7]. An overview of fetal oxygenation patterns in different types of CHD and healthy fetuses is presented in Figure 1.

Optimal functioning of the fetal circulatory system is crucial for fetal brain development as it secures the supply of oxygen and nutrients to the body and brain [18]. Cerebral dysmaturation in the presence of CHD has been argued to originate from the inability of the cardiovascular system to meet the heightened metabolic demands throughout the third trimester of pregnancy [19], a critical period in brain development in which rapid growth, synaptogenesis, neuronal migration, and myelination take place [20, 21]. Animal models demonstrate reduced neurogenesis, altered microglial morphology, and impaired myelin integrity under chronic fetal hypoxia [22, 23, 24]. In fetal CHD, impaired cerebral oxygen and substrate delivery particularly affect the ventricular, intermediate, and subplate zones, containing neural progenitor cells, premyelinating oligodendrocytes, and subplate neurons essential for proliferation, myelination, and cortical organization [25]. At a macroscopic level, diminished cerebral oxygen delivery and consumption have been associated with smaller total brain volume, cortical gray matter volume, and reduced gyrification in fetal CHD [7, 9, 19, 26], persisting into the preoperative neonatal period [27]. Reduced fetal brain volume and cortical development have in turn been associated with increased risk for neonatal ischemic and white matter injury as well as poorer cognitive, language, and motor outcomes at 2 years of age [8, 27, 28, 29]. Findings regarding the neurobiological origin of altered brain development in CHD highlight important potential targets for fetal neuroprotective strategies.

Aside from disruptions in cardiac physiology, placental pathology has also been identified as a contributing factor to adverse brain development in utero [12, 30, 31]. The placenta plays a crucial role in fetal development by mediating oxygen and nutrient exchange [32]. The heart and placenta develop in parallel through shared regulatory pathways involved in vascular development [33]. Therefore, in CHD, both organs may be affected. Several studies have shown associations between CHD and functional and structural placental abnormalities, such as reduced placental oxygenation, maternal vascular malperfusion lesions, preeclampsia, low placental weight, anatomical cord abnormalities, and inflammatory lesions [7, 34, 35, 36, 37, 38, 39, 40, 41, 42]. Placental pathology has been found to be associated with reductions in cortical gray matter, cerebellum, and total brain volume [12]. The growing evidence linking placental abnormalities to adverse brain development emphasizes that the brain in fetal CHD is exposed to multiple stressors [12, 30, 31], further underscoring the need for developing prenatal neuroprotective strategies.

Currently, there is no standardized neuroprotective care regimen for fetuses with CHD. This review provides a comprehensive overview (Table 1) of neuroprotective strategies in fetuses with CHD, including therapeutic strategies, fetal surgery, and modifiable prenatal and perinatal risk factors to optimize fetal brain development, minimize the risk of hypoxic‐ischemic brain injury, and thereby mitigate the progression of neurodevelopmental sequelae.

**TABLE 1:** Overview of clinical studies on neuroprotection in fetal CHD.

Study	N per group	Included CHD subtypes	Gestational range	Intervention	Method	Main findings
Szwast et al. [43]	43 CHD	HLHS	21–40 GW	Acute MH	Oxygen at 8 L/min for ≥ 10 min	Low baseline MCA‐PI compared to z‐scores of healthy fetuses
		HLHS variant				Increased MCA‐PI compared to baseline at ≥ 28 GW
						Baseline MCA‐PI negatively associated with increase of MCA‐PI
Zeng et al. [44]	28 CHD	Borderline left heart	23–37 GW	Acute MH	Oxygen at 8L/min for 15–30 min	Low baseline MCA‐PI compared to HC at ≥ 28 GW
	28 HC					Increased MCA‐PI compared to baseline at ≥ 28 GW
						Decreased vascular flow indices compared to baseline at ≥ 28 GW
						HC showed unchanged MCA‐PI and vascular flow indices compared to baseline
You et al. [45]	30 CHD	AO	22–39 GW	Acute MH	Oxygen at 15 L/min for 6 min	Increased placental oxygenation compared to baseline in all groups
	56 HC	Non‐AO				Greatest increase of placental oxygenation in SVP
		SVP				Increased cerebral oxygenation in SVP and AO compared to baseline
		TV CHD				
Hogan et al. [46]	43 CHD	LSOL	33–35 GW	Acute MH	Oxygen at 8 L/min for ≥ 10 min	LSOL showed lower baseline MCA‐PI z‐scores compared to HC
	17 HC	RSOL				TGA and HC showed increased MCA‐PI z‐scores compared to baseline
		TGA				RSOL showed reduced MCA‐PI z‐scores compared to baseline
Peyvandi et al. [47]	24 CHD	LSOL	33–35 GW	Acute MH	Oxygen at 8 L/min for 5 min	TGA and LSOL showed lower baseline cerebral oxygenation than HC
	20 HC	RSOL				LSOL and HC showed unchanged cerebral oxygenation
		TGA				TGA showed increased cerebral oxygenation compared to baseline
						RSOL was not included in analyses
NCT03944837	170 CHD	SVP	N/A	Acute MH	Oxygen at 10–15 L/min for 30–35 min	N/A
		TV CHD				
		TGA				
NCT03147014	600 CHD	N/A	All GW	Acute MH	Oxygen at 10 L/min for 10–15 min	N/A
NCT03771534	40 CHD	TGA	N/A	Acute MH	Oxygen at 10–15 L/min for < 45 min	N/A
					At MRI and < 30 min at echocardiogram	
Edwards et al. [48]	9 CHD	LHH	26.3–34.3 GW	Chronic MH	Oxygen at 8–9 L/min for 6.5–14.6 h/day over 33–84 days	Low baseline MCA‐PI compared to z‐scores of healthy fetuses
	9 CHD controls					Daily MH duration positively associated with increase of MCA‐PI
						Slower fetal biparietal diameter growth in chronic MH group
						Smaller head circumference at 6 months in chronic MH group
						No difference in neurodevelopment between groups at 6 and 12 months
Xu et al. [49]	23 CHD	LSOL	19.0–41.0 GW	Chronic MH	Oxygen at 6 L/min for 3 h, twice daily	Larger total intracranial volume than CHD controls and smaller than HC, with group differences increasing over time
	23 CHD controls					
						Larger head circumference than CHD controls and smaller than HC
	23 HC					Negative association between total intracranial volume change and baseline MCA‐PI
					
Lee et al. [50]	25 CHD	SVP	20–32 GW	Chronic MH	Oxygen at 3–4 L/min ≤ 24 h/day for 63 days	No difference in fetal growth, MCA‐PI, cerebroplacental ratio, or head circumference between groups
	217 CHD controls					No difference in neonatal brain volume, brain injury, or neurodevelopental outcome at 18 months between groups
					
					
Gaynor et al. [51]	52 CHD	HLHS	24–39 GW	Progesterone	Two vaginal doses of 90 mg/day	No difference in motor, cognitive, and language scales between groups at 18 months (BSID‐III)
	50 CHD controls	TGA				
		Other				
Stegeman et al. [52] NCT04217421	236 CHD	N/A	> 36 GW	Allopurinol	One dose directly post‐partum, one dose 12 h after birth, and three doses perioperatively of 20 mg/kg	N/A
						
					
McElhinney et al. [53]	70 CHD	Aortic stenosis with evolving HLHS	20–31 GW	Fetal cardiac surgery	Fetal aortic valvuloplasty	MCA‐PI lower than population means before intervention
						MCA‐PI remained low at early post‐surgical and late gestation follow‐up
						Normal fetal head circumference
Laraja et al. [54]	69 CHD	Aortic stenosis with evolving HLHS	20–31 GW	Fetal cardiac surgery	Fetal aortic valvuloplasty	Lower scores regarding overall adaptive functioning (ABAS‐II & BASC‐ II), emotional control (BRIEF), communication, motor, problem solving, and personal‐social development (ASQ‐3), and cognitive, language, and motor development (BSID‐III) in childhood compared to normative population, but similar to reported outcomes in historical HLHS cohorts without fetal intervention.

*Note:* Across all studies investigating maternal hyperoxygenation, the fraction of inspired oxygen was set at 100%.

Abbreviations: ABAS‐II: Adaptive Behavior Assessment System Questionnaire‐second Edition; AO: aortic obstruction; ASQ‐3: Ages and Stages Questionnaire‐third Edition; BASC‐II: Behavior Assessment System for Children‐second Edition; BRIEF: Behavior Rating Inventory of Executive Function; BSID‐III: Bayley Scales of Infant and Toddler Development‐third Edition CHD: congenital heart disease; CCHD: critical congenital heart disease; GW: gestational weeks; HC: healthy control; HLHS: hypoplastic left heart syndrome; LSOL: left‐sided obstructive lesion; LHH: left heart hypoplasia; MH: maternal hyperoxygenation; MRI: magnetic resonance imaging; N/A: not available; RSOL: right‐sided obstructive lesion; SVP: single‐ventricle physiology; TGA: transposition of the great arteries; TV: two‐ventricle.

## 2 Neuroprotective Strategies

### 2.1 Maternal Hyperoxygenation

Maternal hyperoxygenation (MH) comprises the administration of oxygen to the mother during pregnancy to alter fetal cardiovascular physiology, either in a single brief session (i.e., acute MH) or multiple longer sessions (i.e., chronic MH) [55]. Recently, MH has received more scientific attention regarding its potential to improve cerebral developmental outcomes of infants with CHD [56]. Those studies hypothesize that in fetuses with compromised cerebral hemodynamics, MH increases the oxygen delivery to the fetal brain by elevating the fetal circulating oxygen tension, thereby stimulating brain growth [7, 46].

Multiple studies have shown varying fetal cerebrovascular responses due to acute MH in CHD [43, 44, 45, 46, 47]. These responses were predominantly reflected by changes in the middle cerebral artery pulsatility index (MCA‐PI), which is an indicator of cerebrovascular resistance [57, 58]. The high variability in oxygen therapy protocols and inclusion of cardiac diagnoses may contribute to the diverse observations across studies (Table 1). The most prominent increases in the MCA‐PI following acute MH were observed in CHD groups with low baseline values of the MCA‐PI, such as TGA and SVP [43, 44, 45, 46]. These specific CHD subtypes are recognized for insufficient cerebral oxygenation in utero [17] and as a result, oxygen supply through MH may result in a substantial elevation in cerebrovascular resistance compared to baseline. Studies investigating fetuses classified as having left‐sided obstructive lesions (LSOL) noted more variability in the response to MH [45, 46, 47]. This may be attributed to the heterogeneity in baseline oxygenation within LSOL, as it includes both single‐ventricular defects (e.g., HLHS) and biventricular defects (e.g., aortic arch anomalies). As for fetuses with right‐sided obstructive lesions, no effects in response to MH have been observed [46].

Increases in the MCA‐PI following acute MH were primarily observed in the third trimester [43, 44]. During this stage, the fetal brain experiences higher metabolic demands to support the rapid volumetric growth and maturation [10], which most likely explains the more distinct effect of acute MH after 28 weeks of gestation [43, 44]. Another contributing factor could be that cerebral autoregulatory mechanisms are less developed in earlier gestational stages, leading to reduced cerebrovascular reactivity to changes in oxygen supply [59].

Chronic MH has been found to improve cardiac development in fetal CHD, indicated by increased dimensions of hypoplastic cardiac structures and the aortic arch, which may in turn improve cerebral substrate delivery [60, 61, 62]. The first study on chronic MH in fetal CHD, examining fetuses with HLHS, raised concerns about the potentially detrimental effects on cerebral development [48]. Although daily MH duration correlated positively with weekly increases in the MCA‐PI, fetuses exposed to MH showed less biparietal diameter growth throughout gestation and smaller head circumferences at 6 months after birth. The authors attributed these findings to MH‐induced placental insufficiency, indicated by insignificantly higher umbilical artery PIs and lower placental weight.

Even so, the decline in cerebral growth could also be caused by the effects of MH on cerebral hemodynamics itself. Increased cerebral oxygen saturation leads to a prolonged state of increased cerebral vascular resistance, resulting in decreased cerebral blood flow [57, 58]. Studies on short‐term MH exposure in fetal sheep and human fetuses with CHD documented this hypothesized impact on cerebral perfusion [56, 63]. While the oxygen demand of the brain may be met through MH, other metabolic demands (e.g., glucose) of the fetal brain may be neglected [64]. Another physiological mechanism that may contribute to reduced cerebral blood flow in response to chronic MH is pulmonary steal. In normal fetal physiology, pulmonary vascular resistance is high, favoring blood flow to the systemic circulation [65]. Hyperoxia, however, has been shown to lower pulmonary arterial resistance by stimulating the production of vasodilatory factors [65, 66]. This decrease in resistance can result in a redistribution of blood toward the pulmonary circulation, reducing flow to the systemic circulation, including the brain. Moreover, multiple neonatal studies have highlighted the susceptibility of the developing brain to hyperoxia related to an underdeveloped capacity in scavenging oxygen radicals [67]. In this context, MH might further affect brain development and white matter maturation, as hyperoxia has been shown to disrupt survival and differentiation of neuronal cells, particularly pre‐oligodendrocytes [67, 68].

It is possible that the high dose of oxygen administered in this first study (8–9 L/min) increased cerebral vascular resistance beyond the point of neurodevelopmental benefit [48]. A study that administered a lower dose of oxygen (6 L/min) reported a positive effect on cerebral growth in fetuses with LSOL, measured by total intracranial volume and head circumference, compared to fetuses with LSOL who did not receive MH [49]. Additionally, an increase in MCA‐PI was observed in fetuses with LSOL between weeks 4 and 12 of MH administration, remaining stable thereafter. The greatest increase in total intracranial growth after MH was observed in fetuses with low baseline MCA‐PI. These results suggest a positive effect of chronic MH on cerebral growth in fetuses with LSOL, yet cerebral volumes remained smaller than those of healthy controls. Lastly, a study administering an even lower dose of oxygen (3–4 L/min) showed no changes in fetal growth, MCA‐PI, cerebroplacental ratio, or head circumference in fetuses with SVP compared with fetuses with SVP that did not receive MH [50]. Likewise, no difference was found between groups regarding neonatal brain volume, preoperative brain injury, mortality, or neurodevelopmental outcome at 18 months. Perhaps, an intermediate dose of oxygen (e.g., 6 L/min) could benefit neurodevelopmental outcome in fetuses with CHD; however, further research is required as the effects of this dose were only examined in fetuses with LSOL.

In summary, although the literature on acute MH suggests that it may provide neurologic benefits to fetuses with low baseline cerebral oxygenation in the third trimester, the current results on chronic MH are conflicting. This emphasizes the need for more extensive research regarding the effects of chronic MH on cerebral development, including different oxygen doses, a wider range of CHD diagnoses, and long‐term neurodevelopmental outcomes.

### 2.2 Progesterone

Progesterone is a placental hormone that plays an important role in pregnancy maintenance. Additionally, it functions as a neurosteroid that stimulates the repair of neural cells during fetal development [69, 70, 71, 72, 73]. There is increasing evidence that progesterone may function as a neuroprotective agent by means of two mechanisms.

Firstly, progesterone administration is hypothesized to induce a change in microglial cells from phenotype M1, a pro‐inflammatory phenotype associated with neurotoxicity, to phenotype M2, an anti‐inflammatory phenotype that promotes tissue restoration [74]. Following prenatal hypoxemia, there is an observed predominance of the M1 microglial phenotype over the M2 phenotype, increasing the neurologic vulnerability to hypoxic injury [24, 74]. It was proposed that the shift to the M1 microglial phenotype after prenatal hypoxia primes microglia to become activated yet uninflamed, until they are exposed to secondary hypoxic‐ischemic events [74]. Consequently, exposure to a second hypoxic stress event would lead to the overproduction of pro‐inflammatory cytokines and subsequent white matter injury. This implies that prenatal progesterone treatment might offer neuroprotection in fetuses with CHD, especially subtypes characterized by low baseline oxygenation, by counteracting the hypoxia‐induced pro‐inflammatory microglial phenotype change. Additionally, it may offer perinatal neuroprotection at secondary hypoxic events, such as the transition from the fetal placental circulation to the neonatal pulmonary circulation at birth.

Secondly, it is thought that the upregulation of progesterone levels, and subsequent increased metabolization to the neurosteroid allopregnanolone protects against cerebral injury following over‐excitation by promoting postsynaptic hyperpolarization [75, 76]. Fetal allopregnanolone upregulation was found to decrease hypoxia‐induced apoptosis and promote remyelination after hypoxic injury in sheep [76, 77]. In human fetuses, progesterone and allopregnanolone precursors are predominantly supplied by the placenta, resulting in a rapid decline after birth [76]. This decline is even more severe in growth‐restricted fetuses who show suboptimal neurosteroid synthesis due to underdevelopment of the adrenal gland. It is possible that adrenal insufficiency contributes to the increased vulnerability to hypoxic‐ischemic neurologic injury in CHD, suggesting a potential benefit of perinatal progesterone treatment.

A recent clinical trial explored the neuroprotective efficacy of vaginal progesterone administration on neurodevelopmental outcomes in human fetuses with CHD [51]. There was no significant treatment benefit regarding neurodevelopmental outcome at 18 months compared with placebo, quantified using the Bayley Scales of Infant and Toddler Development‐III. The results did, however, suggest heterogeneity in treatment effect for motor and language subscales regarding sex and CHD diagnosis. That is, motor and language scores were higher in females, and motor scores were higher in toddlers with CHD diagnoses other than HLHS and TGA receiving progesterone rather than placebo in utero. The authors attributed the difference in treatment effect between males and females to differences in placental and fetal development, rendering males more prone to impaired developmental outcomes. Additionally, it was hypothesized that differences in treatment effects between CHD subtypes are related to differences in placental compensatory mechanisms.

Most importantly, this clinical trial confirms the safety and feasibility of progesterone administration in human fetuses, consistent with prior literature regarding preterm birth prevention [78], paving the way for future clinical trials to further explore the potential neuroprotective effects of fetal progesterone treatment in CHD.

### 2.3 Antioxidants

Antioxidant strategies may help reduce hypoxia‐related oxidative stress in utero through modulation of reactive oxygen species production and enhancement of antioxidant defenses.

Allopurinol, a xanthine oxidase inhibitor, is hypothesized to provide neuroprotection by reducing the formation of reactive oxygen species during reperfusion after hypoxic‐ischemic events [79, 80]. Its neuroprotective efficacy has been suggested in neonates with CHD [81, 82, 83], reducing both neuro‐ and cardiac events in infants with HLHS [81]. Additionally, allopurinol administration was shown to be safe and feasible during pregnancy in two clinical trials concerning suspected fetal hypoxia [84, 85]. Exposed fetuses showed less neuronal injury, exhibited by lower neonatal S100 β levels [84, 85], with a more prominent effect in girls, demonstrated by lower neuroketal concentrations [85]. Although these observations are promising, there is insufficient clinical data on its safety profile in pregnancy with prolonged usage [86]. In addition, it is predominantly aimed at preventing hypoxic‐ischemic cerebral injury; hence, it may provide optimal results when administered perinatally, considering that infants with CHD have an increased risk of hemodynamic instability at birth. To investigate whether this is the case, an ongoing randomized‐controlled trial is aimed at examining neurocardiac properties of allopurinol administration surrounding birth (NCT04217421) (Table 1) [52].

Tetrahydrobiopterin is a required cofactor for nitric oxide synthase and is important for monoamine neurotransmitter biosynthesis and mitochondrial functioning [87, 88]. Its administration is hypothesized to decrease neural vulnerability to hypoxic ischemia through the inhibition of superoxide formation, thereby preventing mitochondrial dysfunction due to peroxynitrite generation [88]. One study examined maternal tetrahydrobiopterin administration in a prenatal hypoxic mouse model of CHD, reporting reduced oligodendrocyte apoptosis and prevention of hypoxia‐induced arrest of myelination [89].

These findings highlight the neuroprotective effects of allopurinol and tetrahydrobiopterin in hypoxic‐ischemic conditions in CHD, yet more research in fetal and neonatal CHD cohorts is required. Additionally, the neuroprotective potential of other antioxidant agents, such as melatonin and vitamin C [90, 91], should be explored in CHD populations.

### 2.4 Surgical Cardiac Interventions in Utero

Fetal cardiac interventions are aimed at preventing the in‐utero progression of CHD and increasing neonatal survival through improvement of cardiac anatomy and function [92, 93]. Fetal aortic valvuloplasty, pulmonary valvuloplasty, and atrial septostomy are the most commonly performed fetal cardiac interventions aimed at preventing progression to HLHS, hypoplastic right heart syndrome, and pulmonary venous hypertension, respectively. In addition to improving fetal cardiac physiology, these procedures may offer indirect neurological benefits through improved cerebral substrate delivery and hemodynamics during a critical period of brain growth.

While the cardiac benefits of fetal cardiac interventions are described, information concerning neurodevelopmental outcomes is limited. To date, only one study has evaluated the neurodevelopmental outcome of children who underwent fetal aortic valvuloplasty using an extensive battery of standardized neurodevelopmental assessments [54]. Children who underwent fetal cardiac intervention showed lower scores in adaptive functioning, executive control, motor, cognitive, and language skills, as well as an increased risk of developmental delay concerning communication, motor skills, problem solving, and personal‐social development compared with the normative population. In a prior study, cerebrovascular hemodynamics were reported to be unchanged after fetal aortic valvuloplasty [53].

Although the beneficial effects of fetal cardiac interventions regarding cardiac physiology and mortality have been described, it remains unclear if and to what extent these procedures may exert neuroprotective effects as current evidence suggests the benefits might not outweigh the adverse effects.

### 2.5 Genetic Risk for Neurodevelopmental Impairment

Brain and cardiac development share molecular pathways, meaning that genetic mutations causing CHD may also disrupt neurodevelopment [94]. Heterozygous de novo variants and rare loss‐of‐function variants in chromatin‐modifying genes have been linked to poorer neurodevelopmental outcomes, including reduced verbal comprehension, working memory, social responsiveness, and increased autism risk in children with CHD [95]. Syndromic conditions commonly associated with CHD and neurodevelopmental impairment, such as 22q11.2 deletion syndrome and Down syndrome, further illustrate this shared genetic vulnerability [96, 97].

Early identification of genetic risk factors for neurodevelopmental delay in CHD may guide tailored neurodevelopmental interventions and help construct the theoretical basis for future fetal gene‐targeted interventions.

### 2.6 Modifiable Risk Factors in the Perinatal Period

To optimize neurodevelopment, children with CHD might benefit from early identification of modifiable fetal and perinatal risk factors for suboptimal brain development and postnatal brain injury.

Firstly, in CHD cohorts, lower gestational age at birth, especially prematurity, is associated with higher rates of pre‐ and postoperative white matter lesions and poorer neurodevelopment [98, 99, 100]. At a lower gestational age, the brain is less mature, which heightens the susceptibility to neurologic injury [27, 98, 101]. In addition, multiple studies have found delivery before 39 weeks to be associated with higher mortality rates and longer duration of postoperative hospital stay [102, 103, 104]. The optimal timing and mode of delivery are frequently discussed for neonates with CHD. Their delivery is often induced either vaginally or by cesarean section to guarantee birth at a tertiary cardiac center [105]. Bonthrone et al. [106] showed a positive association between induced vaginal delivery and preoperative white matter injury. The reason for the induced vaginal delivery, however, was not specified, making it unclear whether the association reflects underlying obstetric risk factors or the delivery mode itself. Notably, infants born via induced vaginal delivery had a lower gestational age at birth than infants born after spontaneous delivery (38.7 vs. 39.7 weeks, respectively). Despite this difference, the inclusion of gestational age in the logistic regression model did not reveal an effect on the occurrence of white matter injury [106]. In the study of Claessens et al. [99], the prevalence of preoperative multifocal neurologic lesions in neonates born via induced vaginal delivery and those born via elective cesarean delivery did not differ. Likewise, Kelly et al. [107] did not report an association between delivery induction and preoperative white matter lesions.

Based on these findings, we suggest that the birth plan of fetuses with CHD should be individually tailored to the clinical needs of the mother and infant. If achievable, the delivery should be postponed to the end of gestation to optimize neurological outcome. However, to prevent unexpected delivery outside the tertiary center, the housing location of parents should be taken into account. That is, parents who live in close proximity could travel to the center when labor is initiated, whereas parents who live further away may require accommodation closer to the tertiary center at the end of gestation.

Compromised maternal and placental health have also been linked to poorer cerebral development in this population [11, 12]. A composite variable of gestational diabetes, gestational hypertension, maternal hypothyroidism, or maternal tobacco use was identified as a risk factor for presurgical white matter injury in infants with severe CHD [11]. In addition, an impaired maternal‐fetal environment has been described to increase the postsurgical risk of death in CHD [108, 109]. Although data on this topic in CHD are still emerging, maternal health conditions such as diabetic, hypertensive, and inflammatory disorders are widely reported to affect fetal brain development outside the context of CHD, with adverse effects on growth, connectivity, and long‐term neurodevelopmental outcomes [110, 111, 112, 113, 114, 115]. Similarly, maternal lifestyle factors such as alcohol exposure, tobacco use, and poor maternal nutritional status have long been recognized for their detrimental impact on neurological outcomes of the fetus [114, 116, 117, 118].

While some maternal risk factors are modifiable, such as lifestyle interventions, tight glycemic control, or the management of pregnancy‐induced hypertension, others are intrinsic and more complex. Nonetheless, proactive monitoring and targeted medical interventions may help decrease the effects of those comorbidities. As for placental dysfunction, emerging evidence indicates that some placental abnormalities may be modifiable. Low‐dose aspirin initiated early in gestation reduces the occurrence of pre‐eclampsia and fetal growth restriction [119]; and may thereby counteract the neurological consequences associated with this condition. In addition, experimental studies show promising results for antenatal administration of melatonin, which may improve placental development and provide neuroprotective benefits to the fetal brain through its anti‐inflammatory, antioxidant, and anti‐apoptotic properties [90, 120]. However, randomized controlled clinical trials during gestation are needed to validate these findings and further explore their potential for improving fetal outcomes [90].

Another maternal risk factor to consider is maternal stress exposure during pregnancy. Mothers carrying a fetus with CHD seem to experience increased psychological distress due to the cardiac diagnosis, including stress, anxiety, and depression [13, 121]. This appears to negatively impact the cerebral development of the fetus, as maternal distress during pregnancies complicated by CHD has been associated with smaller cerebellar and hippocampal volumes [13]. Previous studies in healthy fetuses indicated similar effects, showing associations between prenatal stress and dysfunctional connectivity in the developing brain [122]. Standardized screening for prenatal stress in women with fetal CHD is therefore important as targeted psychotherapeutic interventions could mitigate maternal stress and the subsequent adverse effects on fetal brain development [121].

Overall, these findings highlight the importance of early detection and treatment of maternal risk factors in pregnancies complicated by CHD to mitigate their effects on the fetal brain already at risk for impaired brain development. Possibly, earlier detection of CHD, such as through a first‐trimester anomaly scan [123], may offer the opportunity for closer gestational monitoring and timely interventions.

## 3 Concluding Remarks

Prenatal neuroprotective strategies in children with CHD are warranted as delayed brain development in utero is associated with postnatal brain injury and compromised neurodevelopmental outcomes. This review describes novel neuroprotective interventions before birth and modifiable perinatal risk factors to optimize the neurological outcomes in CHD. Although findings regarding acute MH seemed to reflect increased cerebral oxygenation in fetuses with CHD subtypes characterized by low baseline cerebral oxygenation (i.e., SVP and TGA), results concerning chronic MH remain inconclusive, possibly due to variations in oxygen doses. Therefore, more research is warranted on the optimal duration and dose of MH as well as its benefits for other CHD subtypes with low baseline oxygenation beyond LSOL. The safety and feasibility of progesterone was demonstrated in a recent human fetal trial, with subgroup analyses showing variability in response based on CHD diagnosis and fetal sex, underscoring the need for further investigation into its neurodevelopmental benefits for specific CHD patients. As for perinatal neuroprotection, allopurinol administration surrounding birth seems to be the most promising intervention and is being assessed in an ongoing clinical trial. Fetal cardiac interventions may indirectly improve neurodevelopment through improved cerebral substrate delivery and hemodynamics; however, current evidence does not corroborate this, emphasizing the need for more research.

These potential neuroprotective strategies require more research before they can be implemented in standard care regimens. Until then, to optimize neurologic outcomes, we advise to consider modifiable risk factors in perinatal care, such as gestational age at birth, optimizing maternal and placental health, and mitigating prenatal maternal stress exposure through standardized screening and targeted psychological interventions.

### 3.1 Future Directions

Several strategies for prenatal neuroprotection in fetuses with CHD are currently being researched. To determine the future directions, it is important to delineate the areas that require further exploration. Firstly, although evidence on the impact of the intrauterine environment, such as maternal health, psychological distress, and placental factors, on the fetal brain in CHD is limited, the initial findings underscore that their potential contribution to neurologic deficits needs to be considered [11, 12, 13, 14, 30, 31, 109]. Currently, most placental abnormalities are not considered modifiable, emphasizing the need for further investigation to clarify their role and potential as targets for neuroprotective interventions. A multimodal longitudinal approach combining functional placental magnetic resonance imaging, Doppler ultrasound, structural imaging, and placental histopathology may enhance our understanding of the intrauterine environment. Secondly, determining which CHD subtypes are most at risk of impaired brain development in utero holds the potential for tailored neuroprotection. This ensures that fetuses are not unnecessarily subjected to neuroprotective strategies. However, for this risk‐stratification, large multicenter studies are needed that enable analyses into the variation per cardiac physiology. Thirdly, to determine the optimal timing of prenatal neuroprotection, it is essential to clarify the specific timepoint in gestation where impairments in brain development start. Therefore, evaluation at multiple timepoints with multimodal structural and functional neuroimaging modalities is needed. Fourthly, fetal cardiac interventions, such as aortic or pulmonary valvuloplasty and atrial septostomy, may indirectly benefit neurodevelopment by enhancing cerebral hemodynamics; however, large clinical studies are lacking. Therefore, future research on this topic should examine cerebrovascular hemodynamics, neuroimaging, and neurodevelopmental outcome across various fetal cardiac interventions. Lastly, considering the complex nature of neurodevelopmental issues in CHD, additional avenues for prenatal neuroprotection should be explored, such as genetic, epigenetic, environmental, and socio‐economic aspects.

## Funding

The PhD position of Maaike Nijman was supported by the Dutch Organization for Health Research and Development (ZonMw), project number 848042002, CRUCIAL trial. This subsidizing party did not play a role in writing the manuscript.

## Ethics Statement

The authors have nothing to report.

## Consent

The authors have nothing to report.

## Conflicts of Interest

The authors declare no conflicts of interest.

## Data Availability

Data sharing not applicable to this article as no datasets were generated or analyzed during the current study.
